# Integrative multiomics-histopathology analysis for breast cancer classification

**DOI:** 10.1038/s41523-021-00357-y

**Published:** 2021-11-29

**Authors:** Yasha Ektefaie, William Yuan, Deborah A. Dillon, Nancy U. Lin, Jeffrey A. Golden, Isaac S. Kohane, Kun-Hsing Yu

**Affiliations:** 1 Department of Biomedical Informatics, Harvard Medical School, 10 Shattuck Street, Boston, MA 02115 USA; 2 Department of Pathology, Brigham and Women’s Hospital, 75 Francis Street, Boston, MA 02115 USA; 3 Department of Medicine, Dana-Farber Cancer Institute, 450 Brookline Avenue, Boston, MA 02215 USA; 4 Department of Pathology, Cedars-Sinai Medical Center, 8700 Beverly Blvd, Los Angeles, CA 90048 USA; 5 Burns and Allen Research Institute, Cedars-Sinai Medical Center, 8700 Beverly Blvd, Los Angeles, CA 90048 USA

**Keywords:** Breast cancer, Breast cancer

## Abstract

Histopathologic evaluation of biopsy slides is a critical step in diagnosing and subtyping breast cancers. However, the connections between histology and multi-omics status have never been systematically explored or interpreted. We developed weakly supervised deep learning models over hematoxylin-and-eosin-stained slides to examine the relations between visual morphological signal, clinical subtyping, gene expression, and mutation status in breast cancer. We first designed fully automated models for tumor detection and pathology subtype classification, with the results validated in independent cohorts (area under the receiver operating characteristic curve ≥ 0.950). Using only visual information, our models achieved strong predictive performance in estrogen/progesterone/HER2 receptor status, PAM50 status, and TP53 mutation status. We demonstrated that these models learned lymphocyte-specific morphological signals to identify estrogen receptor status. Examination of the PAM50 cohort revealed a subset of PAM50 genes whose expression reflects cancer morphology. This work demonstrates the utility of deep learning-based image models in both clinical and research regimes, through its ability to uncover connections between visual morphology and genetic statuses.

## Introduction

Breast cancer is the second most prevalent cancer worldwide, with nearly 2 million new cases each year. Histopathologic evaluation of tissue sections, performed by pathologists, is indispensable for the diagnosis and treatment of breast cancer. Evaluation of these slides by human reviewers requires considerable training and expertise, is subject to error, and is time-consuming^1^. Furthermore, visual reviews are limited to known morphology patterns, which does not fully take advantage of the actionable information embedded within histopathologic images^2,3^. Here, we combine deep learning image models from digitalized histopathology slides with transcriptomic analyses to uncover molecular and morphological profiles associated with hormone receptor status and genomic subtypes of breast cancer.

The use of deep learning, a computational technique for learning data representations, to streamline image processing has gradually been gaining acceptance^4,5^. The FDA has recently approved the first screening tool based on neural networks. This algorithm evaluates a retinal image to assess changes of diabetic retinopathy and has been shown to have performance comparable to ophthalmologists^6,7^. In pathology, machine learning has successfully been deployed to differentiate malignant from healthy lung and breast tissue^1,8,9^, stratify lung tumors according to patient prognosis^10,11^, and detect breast cancer micrometastases in lymph nodes^1^.

Essentially all biopsies result in the preparation of histopathology slides to establish the nature and extent of any pathological process. Typically, the preparation of glass slides for interpretation by a pathologist involves tissue fixation, embedding in wax (paraffin), ultra-thin slicing, the mounting of the tissue slices on glass slides and subsequently staining of the tissues for analysis. Slides are often stained with various agents to facilitate the identification of tissue architecture along with cellular and subcellular detail by a human reviewer. The most common protocol, which has been in use for nearly 150 years, utilizes hematoxylin and eosin (H&E), which bind to DNAs and proteins respectively^12^. Other methods, such as immunohistochemistry, involve the use of antibodies which bind specific antigens, permitting identification of specific proteins and other complex molecules^13^. For breast cancer, the allocation of appropriate systemic therapy depends upon accurate and timely assessments of hormone receptor status and HER2 status. This evaluation is complex and not available to patients in all locations, often resulting in inappropriate or incorrect treatments jeopardizing health. Furthermore, emerging data suggest that PAM50 classification status may provide further predictive value with respect to treatment response^14–16^.

In this study, we employ deep learning algorithms on images from H&E stained breast tumors to define two important prognostic and treatment stratifying features; ER/PR/HER2 status and transcriptomic subtypes. We hypothesized that these molecular features would manifest themselves in characteristic histologic patterns on H&E stained slides, and that they could be discerned by machine learning, without the use of accompanying immunohistochemistry-stained slides for ER, PR, or HER2. The ability to successfully classify patient subtypes based on image data implies a morphological basis for the subtype. The identification of recognizable contributing features to an image model suggests that de novo learning of the features was an important step in the process of learning to classify the subtype. We examined the extracted image features associated with the classifications, revealed differential lymphocyte infiltration among patients with different hormone receptor statuses, and connected histopathology patterns with gene expression profiles. Our approaches enable the integration of high-dimensional transcriptomics and histopathology data and facilitate the development of quantitative pathology analyses using deep neural networks.

## Results

### Convolutional neural network (CNN)-based image classifiers can accurately classify histological type and ER/PR/HER2 status of breast cancer patients using only H&E slides

Convolutional neural network-based image classifiers were trained to classify TCGA^17^ BRCA patients (Table 1) based on systematically selected tiles from the corresponding gigapixel, hematoxylin-and-eosin (H&E) stained histopathology images. Of note, the CNN image classifiers were provided only with H&E stained images from frozen tissue sections in TCGA; images of ER, PR, HER2, or other immunostains were not provided. We performed six classification tasks, including (i) tumor vs. normal, (ii) lobular vs. ductal carcinoma histological type, (iii) estrogen receptor (ER) status (positive or negative), (iv) progesterone receptor (PR) status (positive or negative), (v) HER2 receptor status (positive or negative), (vi) PAM50 four-way status (Luminal A, Luminal B, Basal, or HER2 enriched), and (vii) TP53 Mutation status. Consistent with other studies, we were able to attain strong performance in cancer tissue identification^18^, histological types classification, hormone receptor status^19^, and PAM50 status prediction^20^. Our cancer tissue identification and histological types classification results are successfully validated in two independent cohorts with standard formalin-fixed, paraffin-embedded (FFPE) tissue and not frozen sections (AUC ≥ 0.95). The prediction models for TP53 mutation status also achieved high accuracy (0.833 Patient-Level AUC, Table 2). We observed strong image classification performance on tasks traditionally conducted by pathologists using H&E stained slides (tumor vs normal and histological subtype) and tasks that require additional immunohistochemical stains (hormone receptor status) or genomic profiling (PAM50 status). For example, the CNN achieved tile/patient level validation AUCs of 0.929 and 0.982 for ER status, 0.908 and 0.983 for PR status, and 0.829 and 0.979 for HER2 status. These results were all achieved on tissue site-segregated validation cohorts, to simulate the application of a pre-trained model on an external dataset. To examine the ability of genomic and morphological features to inform strong observed CNN performance, we investigated the prediction performance of (i) ER and PR status and (ii) PAM50 status in greater detail.

**Table 1:** Demographics summary of TCGA, Sunnybrook, University of Pennsylvania and the Cancer Institute of New Jersey (UPenn and CINJ) cohorts.

	TCGA	Sunnybrook	UPenn and CINJ
Total number of patients	1099	54	162
Total number of slides	1983	96	279
Total number of image tiles	395116	19200	277524
Average age at diagnosis	59.49	51.23	
Stdev age at diagnosis	13.22	12.33	
% Post menopause	76.05	37.50	
% ER+	77.79	64.29	
% PR+	74.52	53.57	
% HER2+	22.89	24.44	
% Lobular (vs Ductal)	21.58	8.93	0

**Table 2:** Validation ROC-AUC (binary tasks)/accuracy (non-binary tasks) values for all image-based classifiers.

	Image classifier validation set results	
	Patient-level ROC-AUC/accuracy (95% CI)	Tile-level ROC-AUC/accuracy (95% CI)
Tumor vs. normal held-out test set	0.985 (0.968–0.995)	0.921 (0.919–0.924)
Independent validation set	0.950 (0.935–0.964)	0.859 (0.857–0.860)
Histological subtype held-out test set	0.920 (0.845–0.993)	0.800 (0.790–0.803)
Independent validation set	0.996 (0.984–1.0)	0.843 (0.836–0.852)
Estrogen receptor (ER) status +/−	0.982 (0.97–0.994)	0.929 (0.912–0.946)
Progesterone receptor (PR) status +/−	0.983 (0.977–0.989)	0.908 (0.90–0.916)
HER2 Receptor status +/−	0.979 (0.971–0.981)	0.829 (0.80–0.858)
PAM50 Status (4 Class, Top-1)	0.654 (0.636–0.672)	0.406 (0.361–0.451)
PAM50 Status (4 Class, Top-2)	0.790 (0.763–0.817)	0.609 (0.596–0.622)
TP53 Mutation status	0.833 (0.829–0.837)	0.658 (0.634–0.682)

### Immune-related gene ontology terms differ by hormone receptor status (+/−) in breast cancer

To identify potential image-based sources of hormone receptor status signals, gene expression profiles were examined. We do not correlate specific RNA features to image features, but rather use the features important for an RNA-seq based classifier to guide our interpretation of our image-based classifier. Ridge regression conducted over RNA-seq expression profiles of TCGA BRCA patients achieved an AUC of 0.866 for the differentiation of ER receptor status in the unseen test set, and an AUC of 0.827 for the differentiation of PR receptor status using features other than the direct gene expression of the estrogen or progesterone receptor. For reference, a model built on ESR1 expression as a feature alone achieved an AUC of 0.883 toward estrogen receptor status, while a model built on PGR expression alone achieved an AUC of 0.874 toward progesterone receptor status. To identify features important in hormone receptor classification, gene ontology analysis was conducted on the top-ranked genes identified in the regression. The top features for both ER and PR status differentiation included many terms corresponding to the immune function, such as innate immune response, defense response to bacteria, and regulation of STAT protein (Supplementary Table 1, Supplementary Table 2). 56% and 64% of significant gene ontology terms were immune-related for ER and PR status, respectively. For the purposes of differentiating receptor statuses, these results suggested two potential hypotheses: (i) that ER and PR status shared a common signal that was potentially driven by tumor progression or immune infiltration or ii) that signals corresponding to immune infiltration or lymphocyte density might be helpful in distinguishing hormone receptor status. We examined each of these hypotheses in turn.

### CNN image classifiers can distinguish ER (+/−) and PR (+/−) status both independently and jointly

ER and PR status (Table 1) were not distributed independently among the TCGA patients, which is consistent with findings from other cohorts^21,22^. 86.8% of patients had concordant ER/PR status (either both positive or both negative). This finding, combined with the shared immune signal between the ER and PR gene-expression-based classifiers, raised the possibility that the morphological signal utilized by the classifiers was derived from a common source, and that the classifiers had detected this common signal rather than learning ER or PR-specific knowledge. However, this does not appear to be the case. When patients with discordant ER/PR status are examined in isolation, the image classifiers correctly classified these individuals 96.7% of the time. Furthermore, model confidence between concordant and discordant patients of the same label was not found to be significantly different (Table 3). Although the incidence of ER−/PR+ patients was low, the classifiers still achieved high validation set tile-level accuracies. These findings suggest that the original ER/PR classifiers had learned morphological features in the image associated specifically with each receptor status. To further test the performance of the PR classifier, we performed a sensitivity analysis restricted to the ER+/HER2− subcohort. The classifier was able to distinguish between ER+/PR+/HER2-negative versus ER+/PR-/HER2-negative subtypes with tile/patient level validation AUCs of 0.892 and 0.977.

**Table 3:** Comparison of average image-classifier confidence for ER/PR status tasks given concordant (ER+/PR+ or ER−/PR−) or discordant (ER+/PR− or ER−/PR+) patients.

	ER+ (Patient count)	ER− (Patient count)
Concordant	0.775 (118)	0.250 (54)
Discordant	0.784 (14)	0.101 (1)
KS test *P*-val	0.758	0.255
	PR+ (Patient count)	PR− (Patient count)
Concordant	0.728 (118)	0.255 (54)
Discordant	0.803 (1)	0.254 (14)
KS test *P*-val	0.706	0.784

### Lymphocyte morphology have utility in the differentiation of hormone receptor status (+/−)

**Fig. 1:**
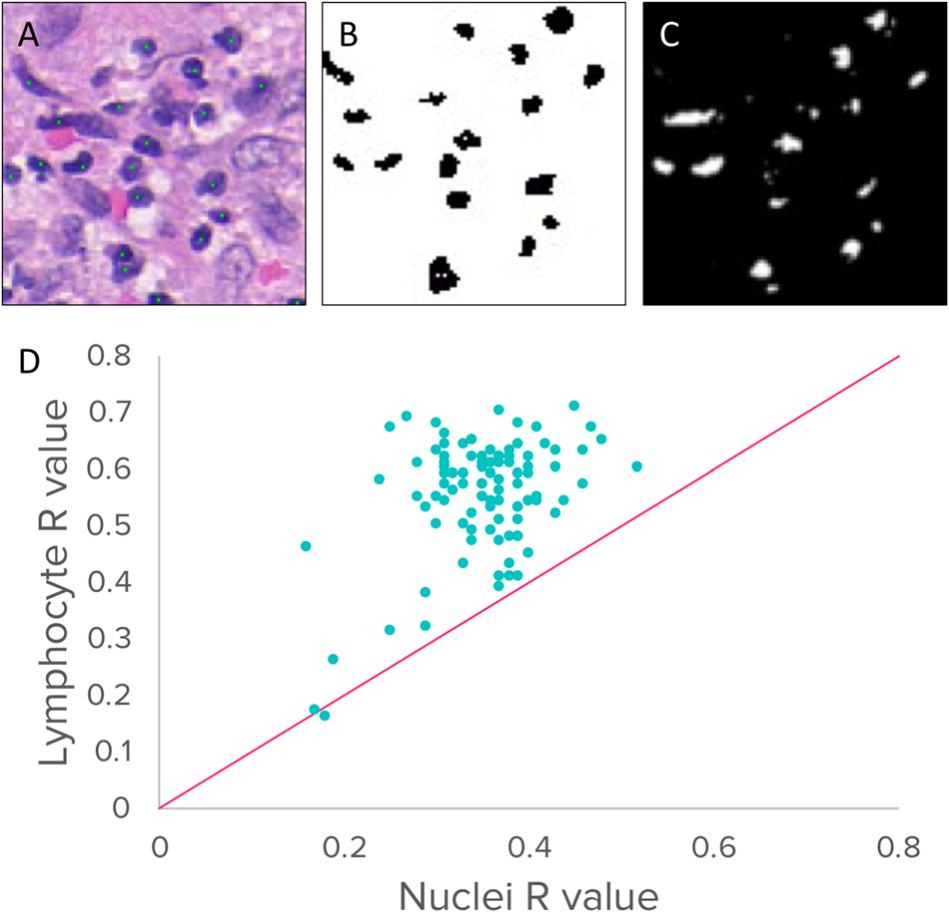
Associations between lymphocytes and convolutional image filters. **A** H&E stained image with annotated lymphocytes, **B** lymphocyte mask, **C** thresholded convolutional activations to 2A, **D** comparison of filter colocalization with lymphocyte and nuclei masks, the line represents equality.

**Fig. 2:**
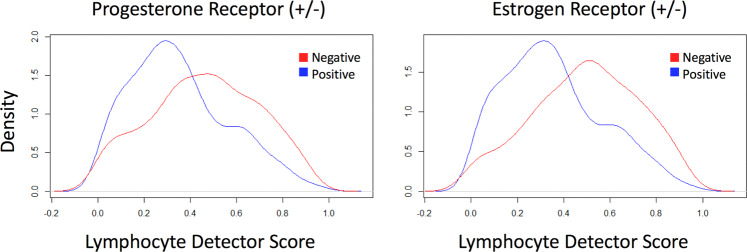
Lymphocyte infiltration patterns distinguished progesterone receptor and estrogen receptor statuses. The distributions of visible lymphocytes for progesterone receptor (left) status + /− and estrogen receptor (right) status +/− are shown.

To examine the hypothesis that visual lymphocyte morphology had utility in hormone receptor status determination, we examined the convolutional activations of layer units in the ER classifier against tiles that had been automatically labeled with a lymphocyte detector (details described in the “lymphocyte detection” section in Supplementary Methods)^23^. The convolutional layer unit activations correspond to the tile regions that the network learned were helpful in making the final determination between receptor status positive and negative. Because units in later network layers are lower resolution due to convolution and pooling steps and are often the result of significant non-linear relationships, we focused our analysis on the first convolutional layer. Layer unit activations were colocalized with lymphocyte-only masks and non-specific nuclei masks to evaluate whether any showed preferential colocalization with lymphocytes over nuclei (Fig. 2A–C). This was found to be the case for one identified filter, where 98% of masks preferentially colocalized with lymphocytes compared to non-specific nuclei (Pearson R vs lymphocyte = −0.553 (*p* < 0.01), Pearson R vs nuclei: −0.354 (*p* < 0.01)) (Fig. 1D). When we removed this filter ER prediction accuracy decreased to 61%. These results suggest that the ER classifier learned morphology resembling lymphocytes as a part of its attempt to distinguish ER+ from ER− receptor status.

To evaluate whether lymphocyte infiltration alone was sufficient to distinguish hormone receptor status, a random-forest-based tile-level lymphocyte detector was trained using CellProfiler^24^ modules and achieved an 85% true positive/true negative accuracy over the pathologist-labeled tileset^23^. This lymphocyte detector was deployed over the TCGA tilesets, and a patient-level lymphocyte-infiltration score was computed, reflecting the average lymphocyte/cell fraction over all tiles associated with the patient. The distribution of these scores between receptor-positive and negative patients was evaluated, along with the distributions of scores for two separate gene-expression-based immune infiltration scores^25,26^. To contextualize the magnitude of any observed effect, and account for the possibility that receptor status and immune infiltration had a common cause of tumor aggressiveness, we evaluated the pathological stage as well (Fig. 2, Table 4). The histopathology-based lymphocyte infiltration score produced more extreme differences between receptor-positive and negative statuses and achieved correspondingly lower *p*-values. In contrast, the pathological stage failed to separate receptor status for either ER or PR. Another predictor of survival, the PAM50 minimal gene set, was also tested for accuracy. The 4-class PAM50 classifier achieved top-1 patient-level validation accuracy of 65.4% and top-2 patient-level validation accuracy of 79.0%, where top-k indicates a correct classification if the true label is in the top-k predictions. Our performance is substantially higher than that of a baseline classifier (25% in a four-class classifier for PAM50 prediction). Further details are provided in Supplementary Results.

**Table 4:** The distinction between receptor status positive/negative patients: comparative performance of image/gene expression-based measurements of immune infiltration for separating receptor status.

	KS Test *p*-value (ER)	KS Test *p*-value (PR)	Bootstrap Δmean *p*-value (ER)	Bootstrap Δmean *p*-value (PR)
Visible lymphocyte signal	<2.2E−16	4.00E−15	<1E−05	<1E−05
ESTIMATE Immune infiltration (Yoshihara, et al.)	5.36E−08	2.14E−03	<1E−05	3.40E−03
TIMER Immune infiltration (Li, et al.)	3.75E−07	1.40E−02	<1E−05	1.43E−02
Pathologic stage (discontinuous distribution)	n/a	n/a	0.2509	0.981

## Discussion

In this work, we have demonstrated the utility of image classification tasks that pathologists currently cannot easily perform. Furthermore, we identified a set of clinical and genomic features whose impact on morphology significantly enhanced the extracted information from H&E stained slides alone. Our study provides new insight into the information content present in biopsy slides and the association between pathology and genomic information. Finally, we demonstrate that deep convolutional neural networks are able to learn in a manner that can be human interpretable. While past work^19,20^ has examined the ability of deep learning models to achieve high accuracy over various breast cancer subtypes, our work attempts to identify which histological or genetic features are utilized by the models. Opening the black box of deep learning models through interpretability is a critical prerequisite to further scientific or clinical integration. We further validated our models on independent external datasets obtained from different sample preparation procedures (frozen tissue vs FFPE section) for two important imaging tasks: cancer detection and subtype identification. Our model’s strong performance in the external datasets after our fine-tuning approach supports the generalizability of these models across different sample preparation protocols.

These results highlight the fact that biopsy slide images are highly information-dense media and that signals present in the image may only be revealed by machine learning analyses. That is, hormone receptor and HER2 expression result in specific morphological features not readily apparent to even a trained pathologist. Furthermore, deconvolution of the convolutional layer filters has the potential to reveal insights regarding gene function.

In contrast, the less optimal performance of image classifiers for genomic features such as PAM50 status or TP53 mutation status emphasizes that the accuracy of such models is highly dependent on the strength of the morphological signals relative to a particular goal. It is impossible for a model based on optical microscopic images to directly observe an indel or frameshift mutation in a particular gene, but it is possible to observe morphological impacts resulting from or related to a given mutation^11,27^. Hence, these models are more likely to find utility in translational systems where the ultimate goal is improving patient diagnosis, prognosis, and treatment stratification, exemplified in this case, by TP53 mutation status, as assessed by an image model, and representing a proxy for the mutation and its causes and consequences, rather than the mutation alone.

Our study contains several limitations. It is difficult to eliminate all of the shared signals between the various classifier tasks due to small sample sizes and the inherent interrelatedness of the phenotypes in question. While we may interpret a particular classifier as differentiating between patients with and without a mutation in a particular gene, it is possible that a proxy feature up- or downstream is responsible for the signals identified by the classifier. This relates to one general weakness of deep learning models: lack of interpretability. Due to the large number of intermediate features in a model and the heavily non-linear relationships between them, the performance of a model can only be understood through examining relationships with known metrics or features. Outside of artificially constructed datasets such as TCGA, the construction of centralized classification models that utilize information from many sites and patients is also difficult due to data privacy and availability issues. Furthermore, it is unclear how generalizable these results would be among populations of different genetic backgrounds. These limitations emphasize the need for the collection of large and diverse datasets to verify the robustness of machine learning models for pathology diagnoses. Although we have successfully validated our models for cancer detection and subtype classification using external cohorts, additional datasets with detailed molecular information are needed to demonstrate the prediction performance of tasks less traditionally associated with morphological signals.

It is important to note that an image-based classifier for an arbitrary feature will not necessarily be successful. The following criteria are all necessary: (i) the presence of sufficient signal in the image relating to the task at hand (e.g., predicting patient ID from the image may not be successful), (ii) presence of sufficient examples of both classes (e.g., alteration of BRCA1 was only present in 4% of our cohort, and survival models in this cohort are impractical due to high rates of early censoring) and (iii) the identified signals from the limited number of training cases are generalizable to the different cohorts. We envision these models used either as hypothesis-generation tools through phenotype screens of centralized datasets, or a tool in a pathologist’s workflow for identifying which patients warrant further examination or testing.

Our work harnesses the recent advancements in CNNs and the availability of clinical, transcriptomic, and histology data to provide a quantitative approach for combining -omics and histopathology analyses. We have shown the ability of image analysis techniques to classify histopathological slides and link the classification to a human interpretable biological motif. This pipeline can be immediately applied to examine the underlying biology of other breast cancer markers. Since the study was conducted retrospectively on a study cohort, further validation of the clinical utility of our models is needed. Our methods are extensible to other cancers, potentially influencing diagnostics and the study of microscopic morphological aberrations in human cancer.

## Methods

### Study cohorts

The objective of this study is to identify the utility of image-based deep learning models toward the differentiation of various clinical and genomic variables in breast cancer. Patient data from BRCA patients was obtained from the TCGA Genomic Data Commons (*n* = 1099), the Hospital of the University of Pennsylvania and the Cancer Institute of New Jersey (HUP-CINJ; *n* = 162)^28^, and Sunnybrook Health Sciences Centre, Toronto, Canada (*n* = 54)^29^. Patients from the TCGA cohort were only included in this study if they possessed a complete set of RNA-seq expression data, image slides, and annotation of the clinical/genomic phenotype of interest. We used frozen section tissue slides from TCGA and verified any selected tiles from the tissue slides contained cancer cells. Phenotypes were treated as annotated, with no imputation or additional interpretation. Image models were initialized from ImageNet weights and stochastically trained three times for each patient-level classification task. ROC-AUC scores of the trained models were first computed in a held-out test partition of the TCGA dataset that did not participate in the training process to assess the internal validity of the models. Two independent cohorts from HUP-CINJ and Sunnybrook were employed to further evaluate the generalizability of our approaches. This study is approved by the Harvard Medical School Institutional Review Board (IRB number: IRB20-0957) and complies with all relevant ethical regulations.

### Model development and training

RNA-seq data, hematoxylin-and-eosin stained tumor tissue slides, and clinical profiles from 1099 breast cancer patients (BRCA) were obtained from the TCGA data portal. Hematoxylin-and-eosin stained histopathology slides and clinical profiles of patients from the independent validation cohorts were also obtained from archived datasets. High-resolution (×20 or ×40) whole-slide images from each TCGA BRCA patient were identified and annotated with the classification task of interest (tumor/normal, histological subtype, ER status, PR status, HER2 status, TP53 mutation status, PAM50 status). The top 200 224 × 224 tiles from each slide were extracted based on RGB pixel density, to account for uneven distributions of tissue across the sliding window^30^. Density was defined as the percentage of non-white pixels (RGB values all less than 200) within the tile window. Tiles were then distributed between train, test, and validation cohorts based on their tissue source site (described below). Cohorts were constructed such that tiles from the same TCGA tissue site did not appear in more than one of the test, training, and validation cohorts. Keras was used to train Inceptionv3 CNN models for each classification task using the created cohorts. Inception v3 was chosen due to its established performance in medical image classification tasks and transfer learning^31,32^. Aggressive regularization, dropout, and image augmentation were utilized during training: images were rotated, shifted, zoomed, and reflected randomly and with random magnitude during training. Model parameters, including learning rate and batch size, were initialized based on lung cancer models were validated on unseen patients. The tile-level classification was re-aggregated to the patient level by averaging over all tiles attributable to a given individual patient (range voting). First-past-the-post voting was evaluated as well and did not have a significant effect on reported accuracy. The area under the receiver operating characteristic curve (AUC) scores were calculated over the patient level for all tasks. To account for the different imaging platforms used in the independent validation sets, fine-tuning on the external datasets was performed by training on a combination of TCGA training data and 10% of data from the new cohorts. The fine-tuned models were tested on patients not included in the training and fine-tuning process in both TCGA and external dataset test sets (Tables 1 and 2). We only perform this fine-tuning and external dataset evaluation for the Tumor vs. Normal and Subtype models. The rest of the models were trained and tested on TCGA data. Further details are provided in Supplementary Materials.

### Reporting summary

Further information on research design is available in the Nature Research Reporting Summary linked to this article.

## Supplementary information

Supplementary Information

Reporting summary

## Data Availability

The data for this study is available from the National Cancer Institute Genomic Data Commons (https://gdc.cancer.gov/). The study cohort can be found under Project TCGA-BRCA. The aggregate data analyzed in this study are available from the corresponding author on reasonable request.

## Code availability

Codes to develop and validate models for this study are available at https://github.com/hms-dbmi/breastCaPathologyTranscriptomics/. There is no access restriction to our GitHub repository.
