# Supplementary material for: Integrative multiomics-histopathology analysis for breast cancer classification
Source: NPJ Breast Cancer. 2021 Nov 29;7:147. doi: 10.1038/s41523-021-00357-y (PMC8630188; doi:10.1038/s41523-021-00357-y)
Supplement: Supplementary file 1 — Supplementary Information [file 41523_2021_357_MOESM1_ESM.pdf]

Supplementary Methods

Tissue Site-based Cohort Definition

Train/test/validation cohorts were constructed according to the following criteria: i) tiles from a given tissue site occur in only one of the three cohorts and ii) no class has more than four times the number of tiles as any other class within a given cohort. Due to differences in mutation, clinical, and PAM50 classification availability between sites, slightly different designations were used for groups of tasks. Three splits were created to reflect the difference in availability: i) tumor/normal, histological subtype and TP53 mutation status differentiation, ii) hormone receptor (ER/PR/HER2) status differentiation, and iii) PAM50 status differentiation.

Training and Finetuning Details For Tumor vs. Normal and Subtype Model

For the Tumor Vs. Normal model and subtype classification model, we separated TCGA slide images into training (80%), validation (10%), and testing (10%) sets. TCGA was separated on the basis of patients, so as to avoid slides of the same patient appearing in multiple data splits. These models were then trained on the training set and tested on the validation and testing sets. Due to the class imbalance inherent in our datasets, we oversampled the training data set to achieve a more equal proportion of positive and negative classes and to make sure our model does not default to the majority class. For finetuning, we took 10% of images in the finetuning dataset, interspersing every 10th batch with a batch of training data. This was to address catastrophic forgetting wherein learning to finetune on the 10% the model would forget features relevant to the training set. More details on the number of images and patients in each split can be found in Supplementary Figure 1.

Hormone Receptor Status Correlations

The estrogen receptor (ER) and progesterone receptor (PR) status of each TCGA and BRCA patient were identified. Only individuals with well-defined (positive or negative) receptor status were considered. For a given receptor type, patients were divided into training and test cohorts in an 80:20 ratio, respectively, such that the ratio of receptor-positive patients to receptor-negative was identical between cohorts. RNA-seq profiles for the training cohort were used to create ridge regression models to differentiate receptor-positive from negative patients. Prior to training, the genes responsible for ER and PR expression were removed (ESR1 and PGR, respectively). ROC-AUC scores of the trained model were computed over the test set. For both ER and PR-based classifiers, the genes most responsible for classification were determined based on ridge coefficient. Gene ontology analysis was conducted over the top 1000 genes found to be significant after FDR correction based on coefficient using the PANTHER Overrepresentation test [1] to identify significant functional motifs. Bonferroni corrections were applied to account for multiple hypothesis testing. Genes corresponding to olfactory receptors were removed due to their known sequence variability. Classifiers trained over the top non-olfactory receptor genes showed similar AUC performance as those trained over all identified genes.

Lymphocyte Detection

A set of tiles with expert-labeled lymphocyte nuclei were used to create a lymphocyte detector. CellProfiler was first used to identify and generate features for nuclei in a non-specific manner, by adapting a pipeline originally created for lung cancer analysis[2]. CellProfiler Analyst was then used to create a Random Forest model to differentiate labeled lymphocytes from other nuclei. This lymphocyte detector was used to analyze TCGA BRCA image tiles and assign each

patient a “visible lymphocyte” score based on average tile-level scores. Patients were scored by computing the proportion of lymphocytes to all cells for all slides from the patient. As a point of comparison, gene-expression-based scores were also computed for each patient using two different scoring methodologies for immune infiltration and lymphocyte density respectively [3,4]. The utility of each scoring method towards differentiation of hormone receptor (ER and PR) status was examined through Kolmogorov–Smirnov testing and bootstrapping difference-of-means. The tumor stage was used as a negative control.

The set of lymphocyte-labeled tiles was also used to examine the interpretability of the CNN-based image classifiers for ER status. The tiles were processed by the classifier, and the sets of network neural activations were identified for each individual tile in the form of layer units. For each tile, lymphocyte masks were created using the expert labels, and nuclei masks were created using the previously generated CellProfiler pipeline. A hard threshold was applied to the convolutional layer units to isolate highly activated pixel regions. These were manually examined for examples that resembled lymphocyte-selective activation. Putative matches were validated by computing colocalization with the lymphocyte and nuclei masks.

PAM50 Analyses

A categorical rather than binary classifier was trained for the differentiation of the four PAM50 subtypes. The output of this model was a vector of four floats that summed to one, which represented the probability of tile assignment to a given subtype, which also was aggregated to the patient level. The confidence and entropy of the classification of a given patient could be evaluated as the maximum value and the log2 Shannon entropy of a 4-bin system with counts corresponding to the class probabilities. The entropy of a subject describes how equivocal the classification of the subject is: class probabilities of (1,0,0,0) correspond to a

minimum entropy of 0, while probabilities of (0.25, 0.25, 0.25, 0.25) correspond to a maximum entropy of 2.

To evaluate the distinction between Luminal A and Luminal B PAM50 subtypes among our cohort, UMAP dimensionality reduction [5] was conducted over all available TCGA RNA-seq expression profiles, reducing 20531 genes to 2 embedded dimensions. The positions of profiles corresponding to the PAM50 validation set were identified in the embedded space. Expression data from patients corresponding to the train/test/validation cohort split of the image classifiers were standardized to unit variance and zero mean and utilized to train a multilayer perceptron deep learning model in Keras and a logistic regression model using Scikit-learn.

UMAP dimensionality reduction was also conducted over the penultimate feature vector of the image classifier which corresponds to a list of 256 features used to make the final class designation. Correlations between the UMAP reduced image features and PAM50 genes were evaluated at the patient level: correlations were deemed significant if they had a correlation coefficient larger in magnitude than +/- 0.33 and a statistically significant p-value, adjusted for multiple hypothesis testing. The coefficients of the PAM50 genes from the logistic regression model were extracted and a ranked list was created, with the top 25 genes designated as ‘important.’

Supplementary Results

Histopathology-based Differentiation of PAM50 Subtypes

Gene expression data has historically been used to classify patients into one of HER2 enriched, Luminal A, Luminal B, or Basal breast cancer subtypes, which have been shown to be useful predictors of survival [6,7]. The PAM50 minimal gene set consists of 50 genes and is commonly used to classify patients in this way [8,9]. Since PAM50 subtypes have been implicated in risk stratification and survival prediction[10], our analyses demonstrated that the extracted histopathology features can inform patient prognosis. PAM50 status represented a significantly more difficult classification task, due to i) the unclear association with histomorphology and ii) the limited available sample size: only 480 patients had both PAM50 clinical designations and RNA-seq expression data in our cohort. The 4-class PAM50 classifier achieved a top-1 patient-level validation set accuracy of 65.4% and top-2 patient-level validation set accuracy of 79.0%, compared to a baseline of 25%, due to the balanced construction of the test and training cohorts (Supplementary Figure 2). First-past-the-post (FPTP), instant runoff, weighted FPTP, and range voting schemes were all tested for tile-to-patient classification aggregation with nearly identical results (Supplementary Table 3). We observed significant (evaluated using KS test) difference in tile-level confidence ($p < 2.2e-16$) and Shannon entropy ($p < 2.2e-16$) between correctly and incorrectly classified tiles. This observation led us to hypothesize that incorrectly classified patients might have a more ambiguous status. A UMAP manifold of all available TCGA BRCA RNA-seq expression data was created and was projected with the validation set (Supplementary Figure 1B). We hypothesized that Luminal A and Luminal B are difficult to distinguish in the UMAP-embedded space with this cohort, even though the embedded space was created with the use of expression data. To evaluate this, multilayer perceptron (MLP) and logistic regression

models were trained over the TCGA expression data using identical patient-level train/test/validation splits as the image classifier. This achieved top-1 validation accuracies of 79.1% and 78% respectively. In both cases, a plurality of errors of these classifiers (52.6% and 50%) came from the inability to distinguish Luminal A and Luminal B. Similarly, 40.9% of the errors made by the image model were due to confusion between Luminal A/B (Supplementary Table 4). The performance of the MLP and logistic regression models can be interpreted as the performance of idealized classifiers for PAM50 subtypes given the sample size/distribution/quality of the available cohort. In other words, the difference in performance between the image models and expression models can be interpreted as gene expression features that arguably do not manifest morphologically.

To examine which features the image model prioritized, correlations between the penultimate feature vector of the image model and gene expression of the PAM50 genes were evaluated (Supplementary Table 5). 25 of the 50 genes had statistically significant adjusted p-values and a correlation coefficient greater than +/- 0.33, suggesting that some morphology feature extraction is associated with expression. When the logistic regression coefficients of the PAM50 genes were considered as well, the genes could be sorted based on both morphological impact and contribution towards classification. The expression levels of 13 PAM50 genes (Supplementary Figure 1C, top left) had comparatively stronger contributions to PAM50 classification, but their known biological functions did not have strong associations with morphological signals, suggesting information relating to their expression or behavior only indirectly associated with the image-based classifier. Gene ontology analysis of the genes most visible to the model found that these genes were highly involved in cell cycle regulation (Supplementary Table 6).

Supplementary Materials

Supplementary Table 1. List of identified GO terms for ER classification.

Supplementary Table 2. List of identified GO terms for PR classification.

Supplementary Table 3. Comparison of PAM50 tile aggregation schemes.

Supplementary Table 4. Error analysis of PAM50 status classifiers.

Supplementary Table 5. Correlation of PAM50 RNA-seq expression with CNN image features.

Supplementary Table 6. List of identified GO terms for the most visible PAM50 genes.

Supplementary Table 7. Performance metrics for image-based classifiers.

Supplementary Table 8. Heterogeneity in tile-level prediction.

Supplementary Figure 1. Data split for tumor vs. normal and histological subtype models.

Supplementary Figure 2. Image-based classification of PAM50 status.

Supplementary Table 1. List of identified GO terms for ER classification.

**Gene Ontology Term**	**Bonferroni Corrected P-value**
cellular component organization or biogenesis (GO:0071840)	2.48E-12
cellular component organization (GO:0016043)	1.18E-10
cellular process (GO:0009987)	1.00E-08
defense response to bacterium (GO:0042742)	3.81E-08
cellular localization (GO:0051641)	8.60E-08
protein localization (GO:0008104)	9.89E-08
localization (GO:0051179)	5.12E-07
macromolecule localization (GO:0033036)	5.57E-07
defense response to other organism (GO:0098542)	7.19E-07
cellular metabolic process (GO:0044237)	8.19E-07
cellular nitrogen compound metabolic process (GO:0034641)	2.88E-06
establishment of localization (GO:0051234)	4.19E-06
defense response to Gram-negative bacterium (GO:0050829)	6.34E-06
cellular component biogenesis (GO:0044085)	6.57E-06
nucleobase-containing compound metabolic process (GO:0006139)	7.79E-06
transport (GO:0006810)	8.98E-06
cellular macromolecule metabolic process (GO:0044260)	1.07E-05
establishment of protein localization (GO:0045184)	1.08E-05

nitrogen compound metabolic process (GO:0006807)	1.30E-05
regulation of receptor activity (GO:0010469)	1.57E-05
macromolecule biosynthetic process (GO:0009059)	1.58E-05
nitrogen compound transport (GO:0071705)	1.65E-05
cellular macromolecule biosynthetic process (GO:0034645)	1.74E-05
intracellular transport (GO:0046907)	1.76E-05
amide transport (GO:0042886)	1.95E-05
metabolic process (GO:0008152)	2.32E-05
gene expression (GO:0010467)	2.99E-05
peptide transport (GO:0015833)	3.07E-05
heterocycle metabolic process (GO:0046483)	3.29E-05
primary metabolic process (GO:0044238)	4.27E-05
cellular aromatic compound metabolic process (GO:0006725)	4.28E-05
establishment of localization in cell (GO:0051649)	4.71E-05
organic substance metabolic process (GO:0071704)	5.06E-05
protein transport (GO:0015031)	5.11E-05
regulation of cellular component organization (GO:0051128)	5.50E-05
organelle organization (GO:0006996)	6.18E-05
cellular biosynthetic process (GO:0044249)	6.35E-05
nucleic acid metabolic process (GO:0090304)	1.76E-04
biosynthetic process (GO:0009058)	2.82E-04
cell cycle (GO:0007049)	3.04E-04
organic substance biosynthetic process (GO:1901576)	3.38E-04

vesicle-mediated transport (GO:0016192)	3.75E-04
macromolecule metabolic process (GO:0043170)	3.95E-04
cellular protein localization (GO:0034613)	4.33E-04
cellular component assembly (GO:0022607)	5.07E-04
organic cyclic compound metabolic process (GO:1901360)	6.55E-04
organic substance transport (GO:0071702)	8.96E-04
RNA metabolic process (GO:0016070)	1.05E-03
cell cycle process (GO:0022402)	1.16E-03
regulation of catabolic process (GO:0009894)	1.43E-03
biological regulation (GO:0065007)	1.60E-03
cellular macromolecule localization (GO:0070727)	1.70E-03
positive regulation of cellular component organization (GO:0051130)	1.74E-03
macromolecule catabolic process (GO:0009057)	2.22E-03
regulation of cellular catabolic process (GO:0031329)	2.84E-03
regulation of cellular process (GO:0050794)	2.94E-03
cellular nitrogen compound biosynthetic process (GO:0044271)	3.75E-03
cellular response to stress (GO:0033554)	3.98E-03
positive regulation of peptidyl-serine phosphorylation of STAT protein (GO:0033141)	4.32E-03
sensory perception of bitter taste (GO:0050913)	4.96E-03
detection of chemical stimulus involved in sensory perception of taste (GO:0050912)	6.04E-03

defense response to Gram-positive bacterium (GO:0050830)	6.33E-03
positive regulation of molecular function (GO:0044093)	7.52E-03
regulation of peptidyl-serine phosphorylation of STAT protein (GO:0033139)	8.09E-03
natural killer cell activation involved in immune response (GO:0002323)	1.08E-02
peptide cross-linking (GO:0018149)	1.19E-02
regulation of biological process (GO:0050789)	1.37E-02
mitotic cell cycle (GO:0000278)	1.43E-02
detection of chemical stimulus involved in sensory perception of bitter taste (GO:0001580)	1.92E-02
RNA processing (GO:0006396)	2.02E-02
innate immune response (GO:0045087)	2.16E-02
defense response (GO:0006952)	2.39E-02
sensory perception of taste (GO:0050909)	2.78E-02
cellular macromolecule catabolic process (GO:0044265)	3.63E-02
mitotic cell cycle process (GO:1903047)	0.0487

Supplementary Table 2. List of identified GO terms for PR classification.

**Gene Ontology Term**	**Bonferroni Corrected P-value**
cellular component organization or biogenesis (GO:0071840)	4.31E-13
cellular component organization (GO:0016043)	2.22E-11
cellular metabolic process (GO:0044237)	3.18E-10
cellular process (GO:0009987)	7.61E-10
metabolic process (GO:0008152)	1.38E-09
nitrogen compound metabolic process (GO:0006807)	3.50E-09
cellular localization (GO:0051641)	2.05E-08
organic substance metabolic process (GO:0071704)	2.53E-08
defense response to other organism (GO:0098542)	1.64E-07
cellular macromolecule metabolic process (GO:0044260)	2.48E-07
primary metabolic process (GO:0044238)	5.19E-07
protein localization (GO:0008104)	6.20E-07
defense response to bacterium (GO:0042742)	8.53E-07
regulation of cellular component organization (GO:0051128)	1.57E-06
establishment of localization in cell (GO:0051649)	1.86E-06
cellular component biogenesis (GO:0044085)	2.40E-06

macromolecule localization (GO:0033036)	2.65E-06
intracellular transport (GO:0046907)	2.84E-06
macromolecule metabolic process (GO:0043170)	4.62E-06
organonitrogen compound metabolic process (GO:1901564)	1.04E-05
positive regulation of peptidyl-serine phosphorylation of STAT protein (GO:0033141)	1.13E-05
amide transport (GO:0042886)	2.20E-05
vesicle-mediated transport (GO:0016192)	2.53E-05
regulation of peptidyl-serine phosphorylation of STAT protein (GO:0033139)	2.57E-05
peptide transport (GO:0015833)	3.44E-05
natural killer cell activation involved in immune response (GO:0002323)	3.76E-05
sensory perception of taste (GO:0050909)	4.47E-05
regulation of receptor activity (GO:0010469)	4.62E-05
protein transport (GO:0015031)	5.72E-05
localization (GO:0051179)	6.06E-05
establishment of protein localization (GO:0045184)	7.17E-05
cellular protein localization (GO:0034613)	8.76E-05
establishment of localization (GO:0051234)	1.29E-04
organelle organization (GO:0006996)	1.88E-04
cellular component assembly (GO:0022607)	2.02E-04

transport (GO:0006810)	2.63E-04
cellular response to stress (GO:0033554)	3.13E-04
positive regulation of cellular component organization (GO:0051130)	3.28E-04
cellular macromolecule localization (GO:0070727)	3.97E-04
sensory perception of bitter taste (GO:0050913)	4.31E-04
detection of chemical stimulus involved in sensory perception of taste (GO:0050912)	5.37E-04
cellular nitrogen compound metabolic process (GO:0034641)	5.81E-04
cellular biosynthetic process (GO:0044249)	6.98E-04
biosynthetic process (GO:0009058)	7.15E-04
cellular protein metabolic process (GO:0044267)	1.10E-03
nitrogen compound transport (GO:0071705)	1.35E-03
cell cycle (GO:0007049)	1.62E-03
detection of chemical stimulus involved in sensory perception of bitter taste (GO:0001580)	1.64E-03
organic substance biosynthetic process (GO:1901576)	1.66E-03
positive regulation of molecular function (GO:0044093)	2.97E-03
organic cyclic compound metabolic process (GO:1901360)	3.04E-03
organic substance transport (GO:0071702)	3.05E-03
B cell proliferation (GO:0042100)	3.18E-03

nucleobase-containing compound metabolic process (GO:0006139)	3.47E-03
cellular aromatic compound metabolic process (GO:0006725)	3.50E-03
symbiosis, encompassing mutualism through parasitism (GO:0044403)	8.98E-03
heterocycle metabolic process (GO:0046483)	9.99E-03
regulation of catabolic process (GO:0009894)	1.16E-02
intracellular protein transport (GO:0006886)	1.21E-02
response to exogenous dsRNA (GO:0043330)	1.22E-02
protein metabolic process (GO:0019538)	1.68E-02
innate immune response (GO:0045087)	1.96E-02
regulation of organelle organization (GO:0033043)	2.17E-02
RNA processing (GO:0006396)	2.18E-02
regulation of cellular catabolic process (GO:0031329)	2.92E-02
biological regulation (GO:0065007)	3.93E-02
cell cycle process (GO:0022402)	4.04E-02
natural killer cell activation (GO:0030101)	4.42E-02
viral process (GO:0016032)	0.0443

Supplementary Table 3. Comparison of PAM50 tile aggregation schemes. FPTP: each tile contributes one vote to the patient classification based on which PAM50 subclass has the highest predicted probability. Range Voting: each tile contributes a fraction of a vote to each subclass based on the predicted probability for each subclass. Instant Runoff: If at the end of vote aggregation, the winning subclass does not have an absolute majority, the tiles voting for the least popular subclass are rescored based on their next highest choice.

	**Accuracy**	**Shannon Entropy (SD)**	**Confidence of Winning Vote (SD)**
**FPTP**	0.645	1.38 (0.4)	0.613 (0.168)
**Range Voting**	0.645	1.94 (0.181)	0.35 (0.06)
**Instant Runoff**	0.645	N/A	0.644 (0.133)

Supplementary Table 4. Error analysis of PAM50 status classifiers. Each column represents a model. The fraction of errors made by that model for a given type of error is shown.

	**Image Classifier**	**Logistic Regression (RNAseq)**	**MLP (RNAseq)**
**Luminal A/Luminal B**	40.90%	50%	52.60%
**Luminal A/Basal**	27.27%	0%	0%
**Luminal A/HER2 Enriched**	4.55%	20%	15.78%
**Luminal B/Basal**	9.10%	5%	5.26%
**Luminal B/HER2 Enriched**	9.10%	20%	21.05%
**HER2 Enriched/Basal**	9.10%	5%	5.26%

Supplementary Table 5. Correlation of PAM50 RNA-seq expression with CNN image features (significant genes only).

Gene	Correlation Coefficient	Corrected p-value
ACTR3B	0.446133	0.027878
ANLN	0.439927	0.034652
BIRC5	0.469683	0.011749
CCNB1	0.506383	0.002679
CDC20	0.530846	0.000906
CENPF	0.57694	9.19E-05
CEP55	-0.532277	0.000848
CXXC5	-0.445358	0.042979
EXO1	0.523664	0.001256
FOXA1	-0.490487	0.021866
GPR160	-0.429642	0.049242
KIF2C	0.525593	0.001152
MAPT	-0.446603	0.027418
MELK	0.575652	9.84E-05
MKI67	-0.449557	0.024682
MLPH	0.439964	0.034607
MYBL2	0.435172	0.040821
NDC80	0.541003	0.000563
NUF2	0.524316	0.00122

ORC6L	0.571434	0.000123
PHGDH	0.459942	0.016926
PTTG1	-0.458026	0.018162
RRM2	-0.496752	0.004014
UBE2C	-0.48579	0.006269
UBE2T	-0.523774	0.00125

Supplementary Table 6. List of identified GO terms for the most visible PAM50 genes.

Gene Ontology Term	Bonferroni Corrected P-value
cell cycle (GO:0007049)	1.63E-12
cell cycle process (GO:0022402)	2.29E-11
mitotic cell cycle process (GO:1903047)	4.00E-11
regulation of chromosome segregation (GO:0051983)	6.32E-11
mitotic cell cycle (GO:0000278)	1.78E-10
M phase (GO:0000279)	3.36E-09
mitotic M phase (GO:0000087)	3.36E-09
regulation of mitotic nuclear division (GO:0007088)	3.54E-09
regulation of nuclear division (GO:0051783)	1.15E-08
regulation of mitotic sister chromatid separation (GO:0010965)	1.31E-08
regulation of chromosome separation (GO:1905818)	2.17E-08
regulation of mitotic sister chromatid segregation (GO:0033047)	3.82E-08
mitotic nuclear division (GO:0140014)	5.20E-08
cell division (GO:0051301)	5.36E-08
mitotic spindle organization (GO:0007052)	8.75E-08
regulation of sister chromatid segregation (GO:0033045)	1.04E-07
anaphase (GO:0051322)	1.07E-07
mitotic anaphase (GO:0000090)	1.07E-07
biological phase (GO:0044848)	1.78E-07
mitotic cell cycle phase (GO:0098763)	1.78E-07
cell cycle phase (GO:0022403)	1.78E-07
microtubule cytoskeleton organization involved in mitosis (GO:1902850)	2.58E-07
nuclear division (GO:0000280)	2.59E-07
organelle fission (GO:0048285)	6.22E-07
regulation of mitotic metaphase/anaphase transition (GO:0030071)	9.07E-07

regulation of metaphase/anaphase transition of cell cycle (GO:1902099)	1.10E-06
spindle organization (GO:0007051)	1.30E-06
regulation of chromosome organization (GO:0033044)	2.54E-06
establishment of chromosome localization (GO:0051303)	3.66E-06
chromosome localization (GO:0050000)	3.96E-06
chromosome segregation (GO:0007059)	8.28E-06
mitotic prometaphase (GO:0000236)	1.07E-05
regulation of mitotic cell cycle (GO:0007346)	2.87E-05
regulation of cell cycle (GO:0051726)	4.66E-05
nuclear chromosome segregation (GO:0098813)	5.50E-05
metaphase plate congression (GO:0051310)	6.98E-05
microtubule cytoskeleton organization (GO:0000226)	7.16E-05
regulation of cell cycle process (GO:0010564)	8.27E-05
establishment of organelle localization (GO:0051656)	1.18E-04
cytoskeleton organization (GO:0007010)	4.06E-04
negative regulation of mitotic sister chromatid separation (GO:2000816)	5.78E-04
negative regulation of chromosome separation (GO:1905819)	6.55E-04
negative regulation of mitotic sister chromatid segregation (GO:0033048)	8.31E-04
microtubule-based process (GO:0007017)	8.92E-04
negative regulation of sister chromatid segregation (GO:0033046)	1.04E-03
negative regulation of chromosome segregation (GO:0051985)	1.16E-03
regulation of organelle organization (GO:0033043)	1.23E-03
mitotic sister chromatid segregation (GO:0000070)	1.71E-03
negative regulation of mitotic nuclear division (GO:0045839)	2.28E-03
mitotic metaphase plate congression (GO:0007080)	2.72E-03
organelle localization (GO:0051640)	3.28E-03

regulation of mitotic cell cycle phase transition (GO:1901990)	4.37E-03
negative regulation of nuclear division (GO:0051784)	4.74E-03
attachment of mitotic spindle microtubules to kinetochore (GO:0051315)	4.95E-03
sister chromatid segregation (GO:0000819)	6.27E-03
regulation of cell cycle phase transition (GO:1901987)	7.65E-03
mitotic cell cycle phase transition (GO:0044772)	7.75E-03
cell cycle phase transition (GO:0044770)	9.12E-03
positive regulation of mitotic cell cycle (GO:0045931)	1.12E-02
organelle organization (GO:0006996)	1.21E-02
kinetochore organization (GO:0051383)	1.80E-02
chromosome organization (GO:0051276)	1.92E-02
negative regulation of cell cycle process (GO:0010948)	1.92E-02
regulation of exit from mitosis (GO:0007096)	2.08E-02
cellular component organization (GO:0016043)	2.52E-02
attachment of spindle microtubules to kinetochore (GO:0008608)	2.73E-02
anaphase-promoting complex-dependent catabolic process (GO:0031145)	2.88E-02
mitotic spindle assembly checkpoint (GO:0007094)	3.94E-02

Supplementary Table 7. Performance metrics for image-based classifiers. Patient-level accuracy, precision, recall, F1 score, sensitivity, and specificity for all relevant image-based classifiers are shown. Threshold decided based on ROC curve for each classifier to maximize sensitivity and specificity.

Model	Precision	Recall	F1 Score	Sensitivity	Specificity
**Tumor vs. Normal Held-out Test Set**	99%	96%	98%	96%	98%
**Tumor vs. Normal Independent Validation Set**	98%	97%	98%	97%	98%
**Histological Subtype Held-out Test Set**	98%	98%	98%	98%	98%
**Histological Subtype Independent Validation Set**	99%	99%	99%	99%	99%
**ER**	96%	98%	97%	98%	94%
**PR**	96%	95%	95%	95%	95%
**HER2**	90%	91%	91%	91%	94%
**TP53 Mutation Status**	86%	66%	75%	66%	95%

Supplementary Table 8. Heterogeneity in tile-level prediction. Every patient slide was sliced into smaller chunks which were then predicted before aggregation to patient-level prediction. Shown are the average and 25th to 75th percentile ranges of standard deviation for the tile-level predictions before aggregation to patient-level prediction for a few of the image-based classifiers.

Model	Standard Deviation (25th-75th percentile)
**Tumor vs. Normal Held-Out Test Set**	0.23 (0.14 – 0.32)
**Histological Subtype Held-Out Test Set**	0.14 (0.08-0.19)
**ER**	0.18 (0.11 – 0.25)
**PR**	0.21 (0.15 – 0.27)
**HER2**	0.23 (0.15 – 0.31)

Supplementary Figure 1. Data split for tumor vs. normal and histological subtype models.

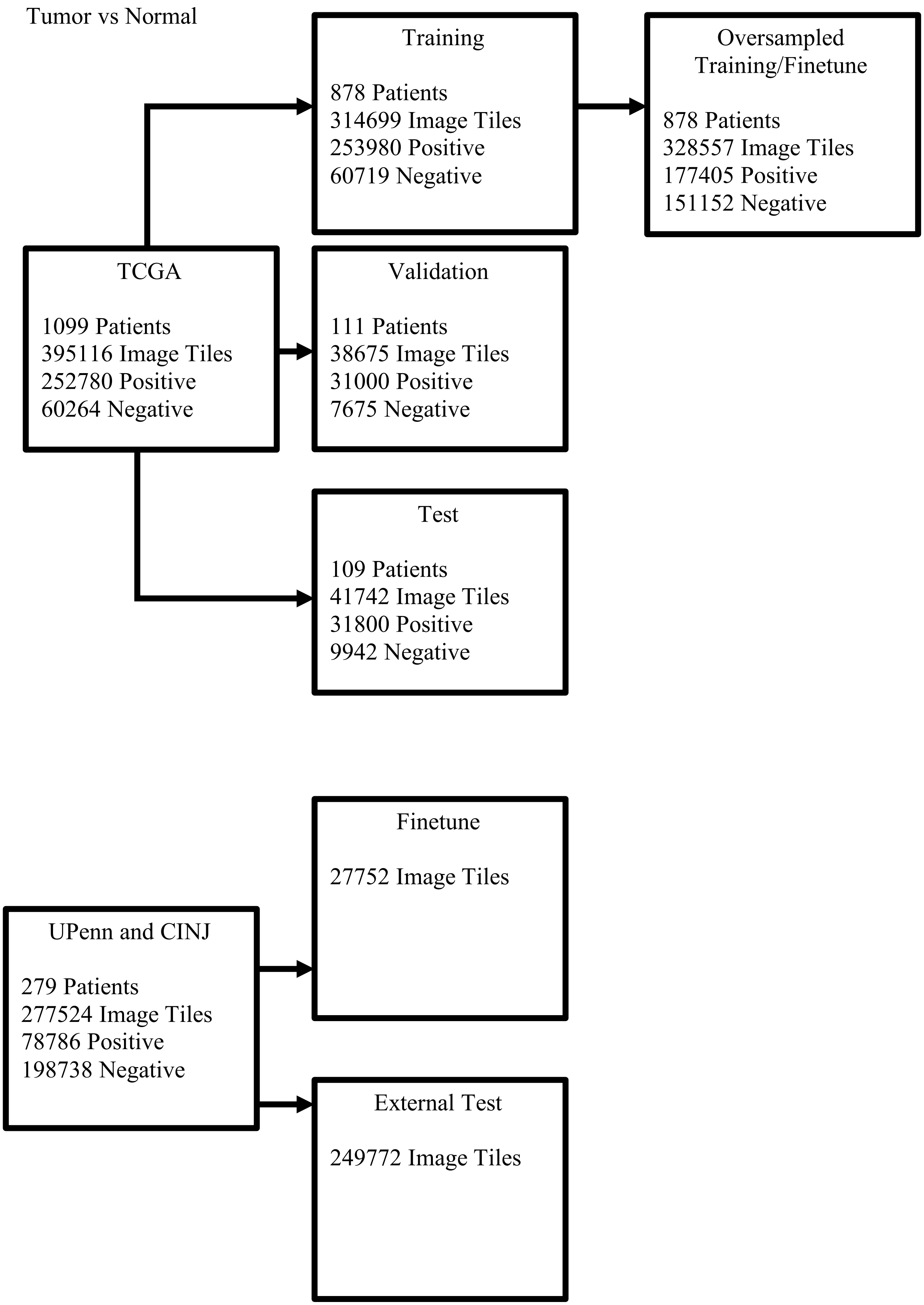

Histological Subtype

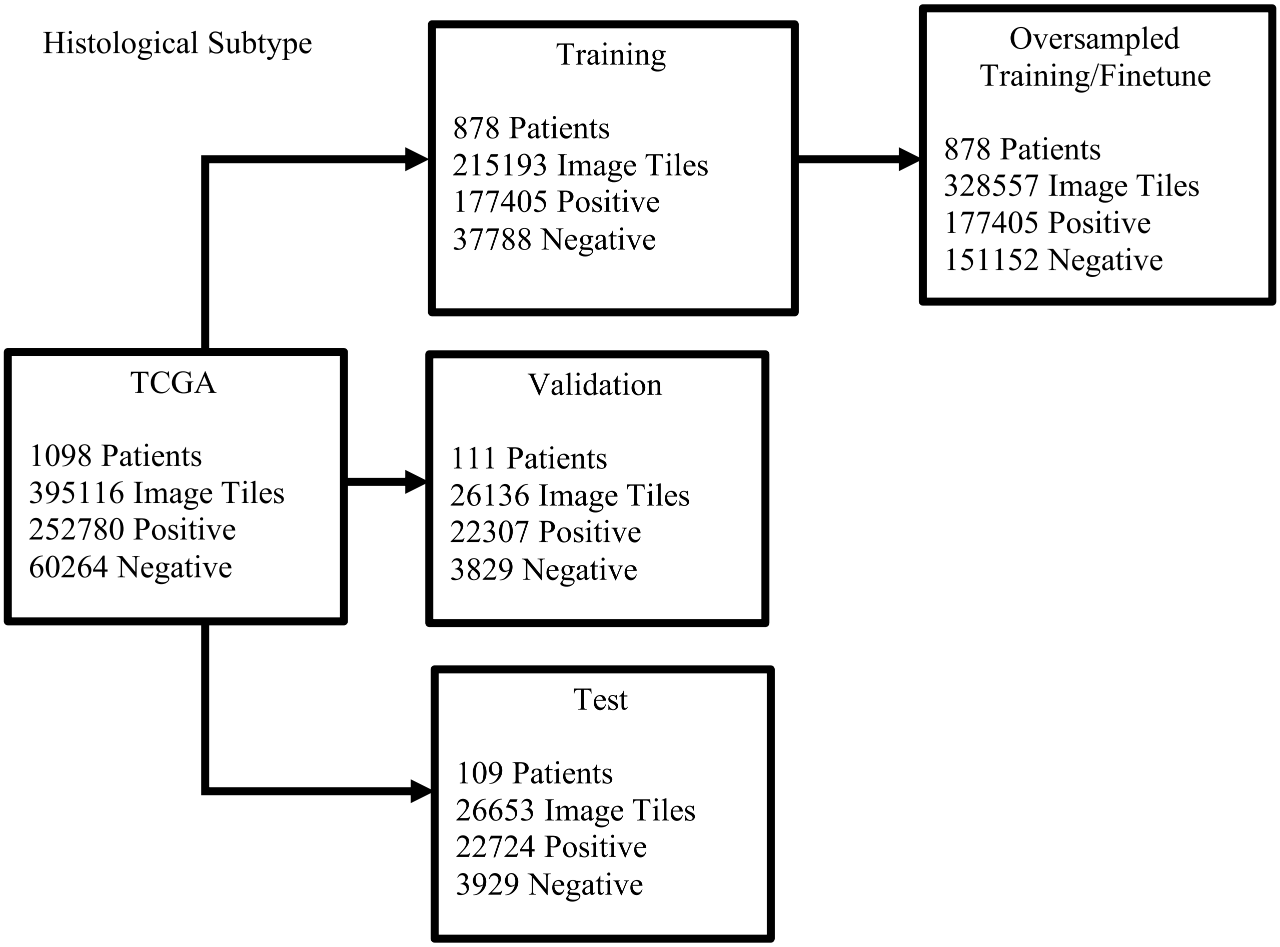

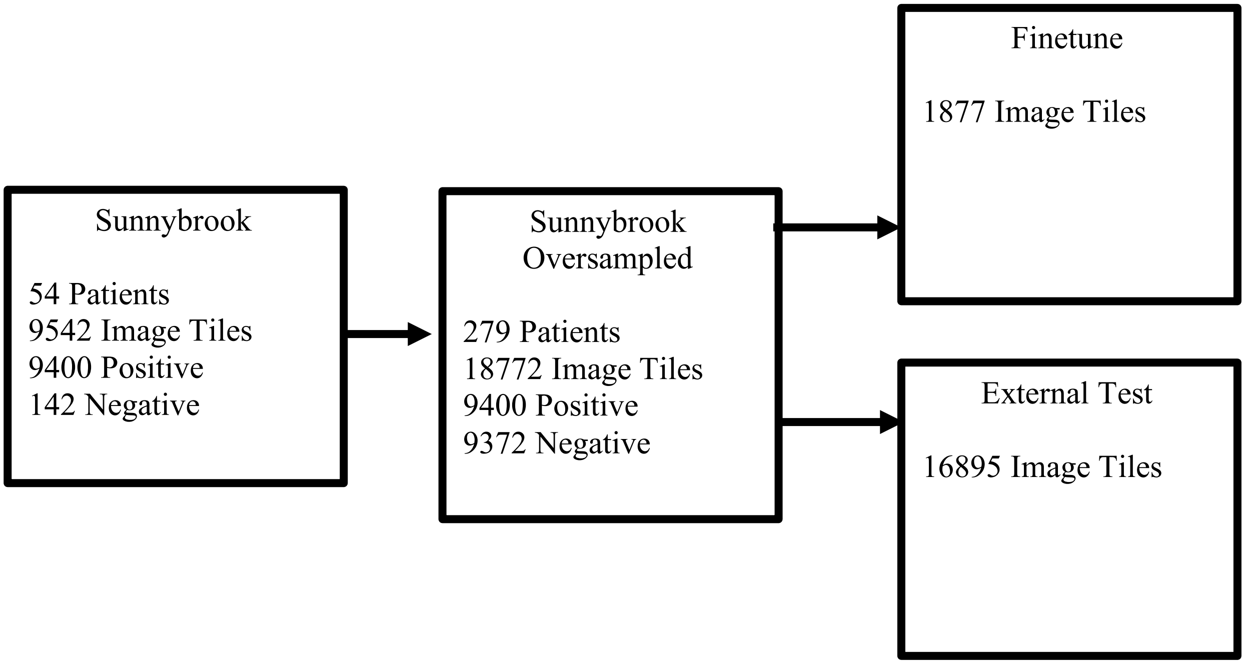

Supplementary Figure 2. Image-based classification of PAM50 status. A) Comparative top-1 accuracy of image classifiers compared to logistic regression/multi-layer perceptron trained over gene expression data. B) UMAP manifold of PAM50 status of the validation cohort. C) PAM50 genes sorted according to visibility/utility in the status determination in the examined cohort.

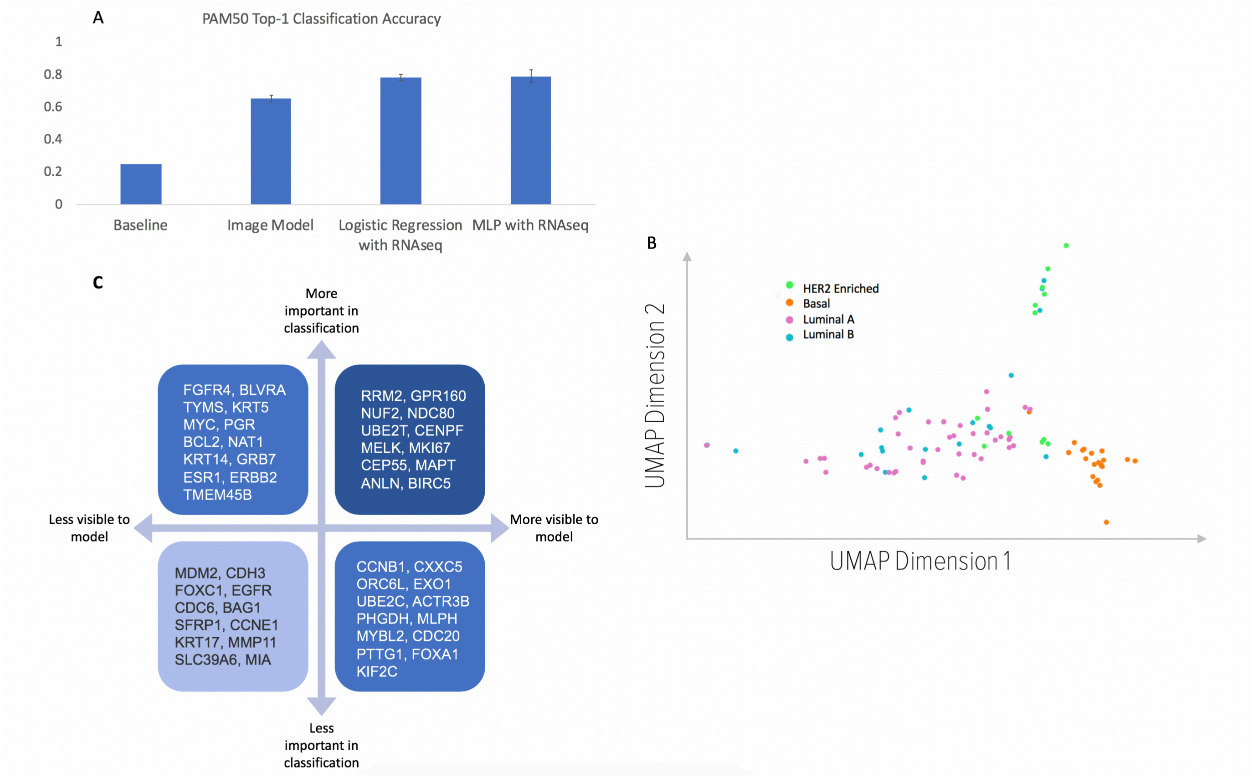

References:

1. Mi, H., Muruganujan, A., Ebert, D., Huang, X. & Thomas, P. D. PANTHER version 14: more genomes, a new PANTHER GO-slim and improvements in enrichment analysis tools. *Nucleic acids research* **47**, D419–D426 (2019).
2. Carpenter, A. E. *et al.* CellProfiler: image analysis software for identifying and quantifying cell phenotypes. *Genome biology* **7**, R100 (2006).
3. Yoshihara, K. *et al.* Inferring tumour purity and stromal and immune cell admixture from expression data. *Nature Communications* **4**, (2013).
4. Li, B. *et al.* Comprehensive analyses of tumor immunity: Implications for cancer immunotherapy. *Genome Biology* **17**, (2016).
5. McInnes, L., Healy, J. & Melville, J. Umap: Uniform manifold approximation and projection for dimension reduction. *arXiv preprint arXiv:1802.03426* (2018).
6. Koboldt, D. C. *et al.* Comprehensive molecular portraits of human breast tumours. *Nature* (2012) doi:10.1038/nature11412.
7. Cheang, M. C. U. *et al.* Responsiveness of intrinsic subtypes to adjuvant anthracycline substitution in the NCIC.CTG MA.5 randomized trial. *Clinical Cancer Research* (2012) doi:10.1158/1078-0432.CCR-11-2956.
8. Bernard, P. S. *et al.* Supervised risk predictor of breast cancer based on intrinsic subtypes. *Journal of Clinical Oncology* **27**, 1160–1167 (2009).
9. Bastien, R. R. *et al.* PAM50 breast cancer subtyping by RT-qPCR and concordance with standard clinical molecular markers. *BMC Medical Genomics* (2012) doi:10.1186/1755-8794-5-44.

10. Pu, M. *et al.* Research-based PAM50 signature and long-term breast cancer survival. *Breast Cancer Res Treat* **179**, 197–206 (2020).
